# Using Andersen’s model of health care utilization to assess factors associated with COVID-19 testing among adults in nine low-and middle-income countries: an online survey

**DOI:** 10.1186/s12913-022-07661-8

**Published:** 2022-02-28

**Authors:** Supa Pengid, Karl Peltzer, Edlaine Faria de Moura Villela, Joseph Nelson Siewe Fodjo, Ching Sin Siau, Won Sun Chen, Suzanna A. Bono, Isareethika Jayasvasti, M. Tasdik Hasan, Rhoda K. Wanyenze, Mina C. Hosseinipour, Housseini Dolo, Philippe Sessou, John D. Ditekemena, Robert Colebunders

**Affiliations:** 1 Department of Health Education and Behavioral Sciences, Faculty of Public Health, Mahidol University, Bangkok, 10400 Thailand; 2 Department of Research Administration and Development, University of Limpopo, Polokwane, South Africa; 3 Department of Psychology, College of Medical and Health Science, Asia University, Taichung, Taiwan; 4 Disease Control Coordination, São Paulo State Health Department, São Paulo, 01246-000 Brazil; 5 Institute of Tropical Pathology and Public Health, Federal University of Goiás, Goiânia, 74690-900 Brazil; 6 Global Health Institute, University of Antwerp, 2000 Antwerp, Belgium; 7 Centre for Community Health Studies (ReaCH), Faculty of Health Sciences, Universiti Kebangsaan Malaysia, 50300 Kuala Lumpur, Malaysia; 8 Department of Health Science and Biostatistics, Faculty of Health, Arts and Design, Swinburne University of Technology, Hawthorn, VIC 3122 Australia; 9 School of Social Science, Universiti Sains Malaysia, 11800 Gelugor, Malaysia; 10 Institute of Nutrition, Mahidol University, Nakhon Pathom, 73170 Thailand; 11 Jeeon Bangladesh Ltd., Dhaka, Bangladesh; 12 Department of Public Health, State University of Bangladesh (SUB), Dhaka, Bangladesh; 13 Department of Primary Care & Mental Health, University of Liverpool, Liverpool, L69 3BX UK; 14 School of Public Health, College of Health Sciences, Makerere University, Kampala, Uganda; 15 University of North Carolina UNC Project Malawi, Lilongwe, Malawi; 16 International Center of Excellence in Research, Faculty of Medicine and OdontoStomatology, Bamako, Mali; 17 Lymphatic Filariasis Research Unit/International Center of Excellence in Research, Bamako, Mali; 18 Research Unit on Communicable Diseases, Polytechnic School of Abomey-Calavi, University of Abomey-Calavi, Cotonou 01, BP 526 Benin; 19 Kinshasa School of Public Health, University of Kinshasa, Kinshasa, 7948 Democratic Republic of the Congo

**Keywords:** COVID-19, testing, adults, LMICs

## Abstract

**Background:**

This study aimed to investigate, using Andersen’s model of health care utilization, factors associated with COVID-19 testing among adults in nine low- and middle- income countries.

**Methods:**

In between 10 December 2020 and 9 February 2021, an online survey was organized in nine low- and middle-income countries. In total 10,183 adults (median age 45 years, interquartile range 33–57 years, range 18–93 years), including 6470 from Brazil, 1738 Malaysia, 1124 Thailand, 230 Bangladesh, 219 DR Congo, 159 Benin, 107 Uganda, 81 Malawi and 55 from Mali participated in the study. COVID-19 testing/infection status was assessed by self-report.

**Results:**

Of the 10,183 participants, 40.3% had ever tested for COVID-19, 7.3% tested positive, and 33.0% tested negative. In an adjusted logistic regression model, predisposing factors (residing in Brazil, postgraduate education), enabling/disabling factors (urban residence, higher perceived economic status, being a student or worker in the health care sector, and moderate or severe psychological distress), and need factors (having at least one chronic condition) increased the odds of COVID-19 testing. Among those who were tested, participants residing in Bangladesh, those who had moderate to severe psychological distress were positively associated with COVID-19 positive diagnosis. Participants who are residing in Malaysia and Thailand, and those who had higher education were negatively associated with a COVID-19 positive diagnosis. Considering all participants, higher perceived economic status, being a student or worker in the health sector, and moderate or severe psychological distress were positively associated with a COVID-19 positive diagnosis, and residing in Malaysia, Thailand or five African countries was negatively associated with a COVID-19 positive diagnosis.

**Conclusion:**

A high rate of COVID-19 testing among adults was reported in nine low-and middle-income countries. However, access to testing needs to be increased in Africa. Moreover, COVID-19 testing programmes need to target persons of lower economic status and education level who are less tested but most at risk for COVID-19 infection.

## Background

In places with ongoing COVID-19 transmission, mass testing (including both symptomatic and asymptomatic individuals) can “help prevent the spread of COVID-19 and identify people in need of care in a timely fashion. A positive test early in the course of the illness enables individuals to isolate themselves thereby reducing the chances of infecting others and allowing them to seek treatment earlier, likely reducing disease severity and the risk of long-term disability, or death.” [1]. A negative test on the other hand does not exempt one from possibly acquiring and transmitting the infection in the future [1]. Therefore, even if an individual is tested negative at a given time point, continuation of safe hygienic measures (washing hands with soaps/using sanitizers frequently, physical distancing, and wearing a face mask) is recommended to protect self and others from COVID-19 [1]. A positive test means isolation is mandatory, and that others with whom the individual may have been in contact since the time of exposure should also get tested [1]. Since nearly half of all SARS-CoV-2 infections are transmitted by pre-symptomatic and asymptomatic people, early identification of infected individuals will play a major role in containing the pandemic [1].

Scanty research has been conducted to identify factors associated with the uptake of COVID-19 testing. In a cross-sectional population-wide survey in Ontario, Canada, of the 758,691 participants who had been tested, 3.3% tested positive for SARS-CoV-2 [2]. The odds of being tested increased with age, male sex, several underlying health conditions, previous use of health care services, and higher household income [2]. Comparing the odds of COVID-19 positive diagnosis, vs having a negative test or being untested, older age, certain comorbidities, such as hypertension, diabetes and heart disease, and higher previous use of health care services were associated with increased odds of a positive SARS-CoV-2 test result [2]. Using UK biobank data, being tested for COVID-19 was associated with male sex, Black ethnicity, lower socioeconomic status, occupation (being a health care worker, unemployed, or retired), comorbidities, exposure to particulate matter (PM) 2.5 absorbance, and ever smoker [3]. Among the tested individuals, only non-White ethnicity, male sex, and lower education were associated with a COVID-19 positive diagnosis [3]. Regarding COVID-19 test uptake/outcome, another important factor to consider is the fact that workers in the healthcare sector stand a higher risk of testing positive. Indeed, a meta-analytic study showed that the pooled prevalence of healthcare workers testing positive for COVID-19 was between 7 to 11% [4], compared to 0.46 to 1.82% in a German population-based cohort study [5]. In a web survey in the Rio de Janeiro metropolitan area, Brazil, in 2020, 42.6% reported ever testing for COVID-19, and the prevalence of a positive PCR results was 16.0%, and of a positive antibody result 10.0%, with no difference across age and comorbidity groups [6].

Health care utilization in terms of COVID-19 testing uptake can be assessed using the Andersen behavioural model explaining that health care utilization is determined by predisposing, enabling/disabling, and needing factors [7]. Predisposing (demographic and social) factors reflect the individuals’ propensity to use health services, enabling/disabling (economic, knowledge) factors are the resources that may facilitate access to services, and the need (health outcome) factors represent potential needs of health service use, such as perceived health status, and chronic conditions [8, 9].

In the current study, we aimed to investigate, using Andersen’s model of health care utilization, factors associated with COVID-19 testing among adults in nine low- and middle- income countries (LMICs) in different phases of the pandemic. Participating countries included Brazil from South America, Malaysia, Bangladesh and Thailand from Southeast Asia and the Democratic Republic of the Congo as the main country from Africa. In nationwide COVID-19 seroprevalence surveys in Brazil the prevalence was 1.9% in May 2020 and 3.1% in June 2020 [10]. In Malaysia, between 16 March 2020 and 31 May 2021, Malaysia’s average 7-day incidence rate was 26.6 reported infections per 100,000 population, and the average test positive ratio and testing rate were 4.3% and 0.8 tests per 1000 population [11]. In a cross-sectional online survey in 2020 in Bangladesh, 315 (15.1%) of 2080 individuals reported to have experienced a COVID-19 infection [12], and in a retrospective cohort study of adult hospitalized patients in 2020 in Thailand, 107 (7.5%) of 1409 patients were COVID-19 infected [13]. In a cross-sectional survey in the general population in Kinshasa, Democratic Republic of the Congo, in 2020, the overall weighted, age-standardized SARS-CoV-2 seroprevalence was 16.6%, and the estimated infection-to-case ratio was 292:1 [14]. Cumulative COVID-19 tests performed per 1000 people were in Brazil 142.47, Malaysia 177.78, Thailand 31.25, Bangladesh 21.12, Uganda 17.27, and Malawi 6.76 on 22 January 2021 [15]. The total number of COVID-19 tests per new confirmed case were on 3 February 2021 in Thailand 111.1, Malaysia 30.3, Bangladesh 6.9, and Malawi 5.7 [16]. COVID-19 vaccine doses administered per 100 people was in Brazil 0.06 on 21 January 2021 [17]. Lack of COVID-19 testing capacity, barriers in accessing COVID-19 testing because of high direct and indirect costs, and stigma associated with COVID-19 testing contribute to insufficient COVID-19 testing in LMICs [18, 19].

## Methods

### Study design, sample, and procedure

This was a descriptive cross-sectional online study conducted in nine LMIC between 10 December 2020 to 9 February 2021. This study was organised by the International Citizen Project (ICP) COVID-19 (ICPCovid) to monitor adherence to COVID-19 prevention measures in low- and middle-income countries [20]. Study countries were selected based on their willingness to participate in the ICP; 50 participants per country was the minimum requirement for enough statistical power [21]. Participant inclusion criteria were being aged 18 years and older, any gender, and provision of electronic informed consent, and exclusion criteria were younger than 18 years and not providing electronic informed consent. Ethical approval was obtained from the Ethics Committees of the participating countries. Further details of the research procedures have been described previously [22]. Briefly, a pre-tested online questionnaire was disseminated via various platforms and consenting respondents submitted their responses via computer/smartphones; all the collected information was stored in a secure server until data extraction and analysis.

### Measures

Using Andersen’s model of health care utilization [7], study variables were categorized into outcome variable, predisposing factors, enabling/disabling factors, and need for care factors.

### Outcome variable

COVID-19 testing/infection status was assessed with the question, “Since the beginning of the COVID-19 outbreak, do you have information on your infection status?” Response options were 1 = not tested/does not know test results, 2 = negative, and 3 = positive.

### Predisposing factors

*Sociodemographic factors* included age (number), sex (male, female), country of residence, educational level (primary, secondary, university undergraduate degree holder, university postgraduate degree holder) and the (estimated) age(s) of their housemate(s) (number: < 12 years, 12–17 years, 18–59 years, and ≥ 60 years).

### COVID-19 preventive measures

Participants were asked, “During the past 7 days, have you been observing any of the following preventive measures against COVID-19? 1) Social distancing of at least 1.5m, 2) Wearing a face mask, 3) Hand hygiene (regular handwashing with soap or using hand gel), and 4) Coughing hygiene (covering the mouth when coughing or sneezing) (Yes/No). A composite non-adherence to all four COVID-19 preventive measure was calculated by coding each negative response with “1”, and otherwise “0″; the composite score (obtained by summing the individual preventive measure’s score for each participant) ranged from 0 to 4 (Cronbach’s alpha 0.7).

### Enabling/disabling factors

Enabling factors included perceived socio-economic status, area of residence (rural/village, sub-urban setting or urban slum, and urban setting/city/town), being a student or worker in the health care sector, and most trusted source of COVID-19 information/advice (whereby the response categories ‘other’, including family and friends, ‘radio/TV’, ‘social media’, and ‘religious authorities’ were all coded as “0”, and ‘health personnel’ coded as 1). Perceived socio-economic status was sourced from the question, “Which of the following categories best describes your current socio-economic situation? Low-income category, lower-middle income category, upper-middle income category, and high-income category.” Correct COVID-19 knowledge was defined as all three affirmative responses to “(1) if there is a possibility of being reinfected after recovering from a previous COVID-19 infection (Yes/No); (2) if COVID-19 infection could be prevented by a vaccine (Yes/No); and (3) if there is currently an effective vaccine against COVID-19 (Yes/No).” [22].

Disabling factors included the assessment of psychological distress with the Patient Health Questionnaire (PHQ-4) for Depression and Anxiety symptoms [23, 24]. The PHQ-4 is a valid and reliable measure of depression and anxiety symptoms in the general population. PHQ-4 is a 4-item inventory rated on a four-point Likert scale. The severity of psychological distress was categorized as “normal (0-2), mild (3-5), moderate (6-8) and severe (9-12) based on the PHQ-4 scores” [23].

*Need for care factors* included three questions on 1) the level of fear/worry of being infected with COVID-19 (ranging from 1 = not at all worried to 5 = extremely worried), 2) having been quarantined (either at home or elsewhere) at any point in time during the COVID-19 epidemic, and 3) chronic/underlying diseases including heart disease, hypertension, diabetes, cancer, HIV, tuberculosis, and chronic asthma; coded as “0” none and “1” at least presence of one clinically diagnosed condition.

### Data analysis

Survey weights were used to control the proportion contribution of each participating country to the total population (aged 15 and above in each country in 2019) estimate. The country-specific weight was used to adjust for any deviations in the estimates due to bias arising from over- and under-coverage. More details about the survey weights were provided previously [22].

Descriptive statistics were used to describe the study population. Logistic regression was used to assess associations between predisposing factors, enabling and disabling factors, need of care factors and COVID-19 testing status, COVID-19 positive versus negative status and COVID-19 positive versus negative and not tested status. Variables significant at < 0.05 in univariate analyses were subsequently included in the multivariable logistic regression models. *P*-values at < 0.05 were considered significant. Statistical analyses were conducted using “STATA software version 15.0” (Stata Corporation, College Station, Texas, USA).

## Results

In total, 10,183 participants were included in the study (6470 Brazil, 1738 Malaysia, 1124 Thailand, 230 Bangladesh, 219 Democratic Republic of Congo (DRC), 159 Benin, 107 Uganda, 81 Malawi and 55 Mali), with median age 45 years, interquartile range 33–57 years, range 18–93 years. Of these, 40.3% knew their COVID-19 infection status during the ongoing pandemic: 7.3% had tested positive and 33.0% had tested negative.

### Predisposing factors

One in five participants (20.2%) were 60 years and older, 64.9% were female, 90.8% lived with other people, 47.5% had a postgraduate education, and 47.8% did not adhere to the four COVID-19 preventive measures.

### Enabling/disabling factors

Most participants (80.4%) resided in urban areas, 48.0% rated their socio-economic status as upper middle to high-income, and 34.4% were students or workers in health care. The most trusted source of COVID-19 information/advice was health care personnel (61.7%); 51.5% had correct COVID-19 knowledge, and 18.5% reported moderate to severe psychological distress.

### Need for care factors

More than half of the participants (51.7%) were very or extremely worried about being (re)infected with COVID-19, 38.2% had been quarantined during the COVID-19 pandemic, and 29.2% had at least one diagnosed chronic/underlying disease (see Table 1).

**Table 1:** Sample and COVID-19 testing characteristics of adults in nine low-and middle-income countries, 2020, 2021

Variable	Sample	COVID-19 testing/infection status		
		Not tested/no results	Negative	Positive
	N (%)	N (%)	N (%)	N (%)
**All**	10,183	6078 (59.7)	3362 (33.0)	743 (7.3)
**Predisposing factors**				
Country				
Brazil	6470 (63.5)	3283 (50.7)	2526 (39.0)	61 (10.2)
Malaysia	1738 (17.1)	1246 (71.7)	473 (27.2)	19 (1.1)
Thailand	1124 (11.0)	1017 (90.5)	104 (9.3)	3 (0.3)
Bangladesh	230 (2.3)	133 (57.8)	60 (26.1)	37 (16.1)
5 African countries	621 (6.1)	399 (64.3)	199 (32.0)	23 (3.7)^a^
Age in years				
18–39	4041 (39.7)	2397 (59.3)	1343 (33.2)	301 (7.4)
40–59	4081 (40.1)	2367 (58.0)	1408 (34.5)	306 (7.5)
60 or more	2061 (20.2)	1314 (63.8)	611 (29.6)	136 (6.6)
Sex				
Male	3579 (35.1)	2164 (60.5)	1178 (32.9)	237 (6.6)
Female	6604 (64.9)	3914 (59.3)	2184 (33.1)	506 (7.7)
Living status				
Lives alone	932 (9.2)	528 (56.7)	329 (35.3)	75 (8.0)
Lives with other people	9251 (90.8)	5550 (60.0)	3033 (32.8)	668 (7.2)
Education				
Primary/secondary	1316 (13.0)	838 (67.7)	315 (25.4)	85 (6.9)
Undergraduate	4028 (39.5)	2612 (64.8)	1163 (28.9)	253 (6.3)
Postgraduate	4839 (47.5)	2567 (53.0)	1873 (38.7)	399 (8.2)
*Preventive measures*				
Not social distancing	3378 (33.2)	1910 (56.5)	1124 (33.3)	344 (10.2)
Not wearing face mask	413 (4.1)	231 (55.9)	136 (32.9)	46 (11.1)
No hand hygiene	1154 (11.3)	680 (58.9)	356 (30.8)	118 (10.2)
No coughing hygiene	3372 (33.1)	1986 (58.9)	1112 (33.0)	274 (8.1)
Not all four measures	4872 (47.8)	2879 (47.4)	1570 (46.7)	423 (56.9)
**Enabling/disabling factors**				
Residence				
Rural	812 (8.0)	654 (80.5)	145 (17.9)	13 (1.6)
Suburban/urban slum	1185 (11.6)	776 (65.5)	334 (28.2)	75 (6.3)
Urban	8186 (80.4)	4648 (56.8)	2883 (35.2)	655 (8.0)
Income				
Low/lower middle	5298 (52.0)	3522 (66.5)	1450 (27.4)	326 (6.2)
Upper middle/high	4885 (48.0)	2556 (52.3)	1912 (39.1)	417 (8.5)
Student/worker in healthcare	3500 (34.4)	1780 (50.9)	1414 (40.4)	306 (8.7)
Most trusted source of COVID-19 information/advice				
Other	3905 (38.3)	2444 (62.6)	1217 (31.2)	244 (6.2)
Health care personnel	6278 (61.7)	3634 (57.9)	2145 (34.2)	499 (7.9)
COVID-19 correct knowledge	5145 (51.5)	2885 (55.0)	1932 (36.8)	428 (8.2)
Psychological distress				
None	4774 (46.9)	3057 (64.0)	1442 (30.2)	275 (5.8)
Mild	3528 (34.6)	2010 (57.0)	1244 (35.3)	274 (7.8)
Moderate	1152 (11.3)	618 (53.6)	418 (36.3)	116 (10.1)
Severe	729 (7.2)	393 (53.9)	258 (35.4)	78 (10.7)
**Need for care factors**				
Worry/fear about being (re) infected with COVID-19 (very or extremely)	5160 (51.7)	3074 (58.4)	1818 (34.6)	368 (7.0)
Has been quarantined during COVID-19 epidemic	3889 (38.2)	1576 (40.4)	1656 (42.6)	657 (16.9)
Chronic condition(s) total	2974 (29.2)	1683 (56.6)	1072 (36.0)	219 (7.4)
Heart disease	419 (4.1)	248 (59.2)	144 (34.4)	27 (6.4)
Hypertension	1825 (17.9)	1053 (57.7)	648 (35.6)	123 (6.7)
Diabetes	694 (6.8)	425 (61.2)	219 (31.6)	50 (7.2)
Cancer	157 (1.5)	80 (51.0)	64 (40.8)	13 (8.3)
HIV	56 (0.5)	26 (46.4)	25 (44.6)	5 (8.9)
Tuberculosis	14 (0.1)	6 (42.9)	5 (35.7)	3 (21.4)
Asthma	730 (7.2)	375 (51.4)	295 (40.4)	60 (8.2)

^a^Ranging from 2.5% in Benin and Malawi to 7.3% in Mali

### Associations with COVID-19 testing

In the adjusted logistic regression model, predisposing factors (residing in Brazil, having attained postgraduate education), enabling/disabling factors (urban residence, higher perceived economic status, being a student or worker in the health care sector, and reporting moderate or severe psychological distress), and need factors (having at least one chronic condition) increased the odds of COVID-19 testing. In addition, in the unadjusted analysis, correct COVID-19 knowledge and worry or fear of being (re)infected with COVID-19 were positively associated with COVID-19 testing status (Table 2).

**Table 2:** Associations with COVID-19 testing and COVID-19 diagnosis

Variable	Odds of COVID-19 testing (95% CI)		Odds of COVID-19 diagnosis, among all individuals tested (95% CI)		Odds of COVID-19 diagnosis, among all individuals tested or not tested (95% CI)	
	Unadjusted	Adjusted	Unadjusted	Adjusted	Unadjusted	Adjusted
**Predisposing factors**						
Country						
Brazil	1 (Reference)	1 (Reference)	1 (Reference)	1 (Reference)	1 (Reference)	1 (Reference)
Malaysia	0.41 (0.36, 0.46)***	0.51 (0.44, 0.59)***	0.15 (0.10, 0.24)***	0.15 (0.09, 0.24)***	0.10 (0.06, 0.15)***	0.13 (0.08, 0.20)***
Thailand	0.11 (0.09, 13.3)***	0.14 (0.11, 0.18)***	0.11 (0.03, 0.35)***	0.14 (0.04, 0.44)***	0.02 (0.01, 0.07)***	0.04 (0.01, 0.11)***
Bangladesh	0.75 (0.58, 0.98)*	0.62 (0.46, 0.83)**	2.36 (1.55, 3.58)***	1.98 (1.24, 3.16)**	1.68 (1.17, 2.42)**	1.41 (0.94, 2.13)
5 African countries	0.57 (0.48, 0.68)***	0.53 (0.53, 0.88)**	0.44 (0.28, 0.68)***	0.52 (0.31, 0.86)	0.34 (0.22, 0.52)***	0.37 (0.22, 0.64)***
Age in years						
18–39	1 (Reference)	–	1 (Reference)	1 (Reference)	1 (Reference)	1 (Reference)
40–59	1.09 (0.93, 1.27)		0.60 (0.43, 0.83)**	0.98 (0.68, 1.40)	0.68 (0.50, 0.93)*	1.16 (0.81, 1.64)
60 or more	1.04 (0.87, 1.23)		0.61 (0.44, 0.86)**	0.89 (0.60, 1.34)	0.67 (0.50, 0.92)*	0.85 (0.60, 1.21)
Sex						
Male	1 (Reference)	–	1 (Reference)	–	1 (Reference)	1 (Reference)
Female	1.07 (0.91, 1.26)		1.43 (0.99, 2.06)		1.42 (1.00, 2.00)*	1.06 (0.73, 1.53)
Living status						
Lives alone	1 (Reference)	–	1 (Reference)	–	1 (Reference)	–
Lives with other people	1.00 (0.82, 1.22)		1.32 (0.92, 1.90)		1.27 (0.90, 1.79)	
Education						
Primary/secondary	1 (Reference)	1 (Reference)	1 (Reference)	1 (Reference)	1 (Reference)	–
Undergraduate	1.22 (0.92, 1.61)	1.17 (0.87, 1.56)	0.57 (0.31, 1.03)	0.53 (0.30, 0.94)*	0.74 (0.42, 1.31)	
Postgraduate	2.01 (1.53, 2.64)***	1.48 (1.10, 1.99)**	0.43 (0.24, 0.75)**	0.41 (0.27, 0.72)**	0.81 (0.47, 1.40)	
Not adhering to 4 COVID-19 preventive measures	0.88 (0.75, 1.06)	–	1.24 (0.87, 1.76)	–	1.27 (0.90, 1.79)	–
**Enabling/disabling factors**						
Residence						
Rural	1 (Reference)	1 (Reference)	1 (Reference)	1 (Reference)	1 (Reference)	1 (Reference)
Suburban/urban slum	2.74 (1.95, 3.84)***	1.25 (0.86, 1.84)	3.83 (1.65, 8.87)**	1.65 (0.72, 3.83)	7.50 (3.38, 16.66)***	2.28 (0.98, 5.35)
Urban	4.66 (3.61, 6.01)***	1.89 (1.40, 2.57)***	3.85 (1.99, 7.44)***	1.60 (0.82, 3.09)	10.90 (5.84, 20.36)***	3.11 (1.57, 6.15)***
Income						
Low/lower middle	1 (Reference)	1 (Reference)	1 (Reference)	1 (Reference)	1 (Reference)	1 (Reference)
Upper middle/high	2.14 (1.82, 2.52)***	1.49 (1.24, 1.79)***	1.59 (1.14, 2.23)**	1.20 (0.85, 1.70)	2.47 (1.80, 3.41)***	1.59 (1.15, 2.19)**
Student or worker in the healthcare sector	1.57 (1.34, 1.85)***	2.05 (1.70, 2.48)***	1.37 (0.98, 1.91)	–	1.77 (1.29, 2.42)***	1.93 (1.37, 2.72)***
Most trusted source of COVID-19 information/advice						
Other	1 (Reference)	–	1 (Reference)	–	1 (Reference)	–
Health care personnel	1.07 (0.91, 1.27)		1.32 (0.91, 1.91)		1.33 (0.93, 1.89)	
COVID-19 correct knowledge	1.36 (1.15, 1.58)***	0.95 (0.78, 1.16)	0.86 (0.61, 1.20)	–	1/07 (0.78, 1.48)	–
Psychological distress						
None	1 (Reference)	1 (Reference)	1 (Reference)	1 (Reference)	1 (Reference)	1 (Reference)
Mild	1.26 (1.06, 1.50)**	1.05 (0.85, 1.29)	1.17 (0.80, 1.73)	1.11 (0.73, 1.71)	1.35 (0.93, 1.95)	1.07 (0.71, 1.59)
Moderate	1.82 (1.37, 2.42)***	1.40 (1.03, 1.91)*	2.57 (1.51, 4.36)***	1.98 (1.16, 3.39)*	3.24 (1.97, 5.33)***	1.22 (1.32, 3.74)**
Severe	1.63 (1.15, 2.32)**	1.40 (1.00, 1.98)*	3.21 (1.75, 5.88)***	2.33 (1.28, 4.25)**	3.58 (2.02, 6.34)***	2.58 (1.45, 4.60)***
**Need for care factors**						
Worry/fear about being (re) infected with COVID-19	1.08 (1.02, 1.15)*	0.97 (0.89, 1.05)	1.03 (0.90, 1.17)	–	1.08 (0.96, 1.22)	–
Chronic condition(s)	1.44 (1.21, 1.72)***	1.25 (1.02, 1.52)*	0.95 (0.64, 1.39)	–	1.21 (0.84, 1.75)	–

*CI* Confidence Interval; ***p < 0.001; ** *p* < 0.01; * *p* < 0.05

Regarding the odds of COVID-19 positive diagnosis among all participants who reported COVID-19 test results in the adjusted analysis, residing in Bangladesh, and moderate to severe psychological distress were positively associated with COVID-19 positive diagnosis. Meanwhile, residing in Malaysia or Thailand and a higher education level were negatively associated with a COVID-19 positive diagnosis. In addition, in the unadjusted analysis, older age, urban residence, and higher perceived economic status were also associated with COVID-19 positivity (Table 2).

Adjusted logistic regression of the odds of a COVID-19 positive diagnosis among all individuals tested or not tested found that urban residence, higher perceived economic status, being a student or worker in the health sector, and moderate or severe psychological distress were positively associated with a COVID-19 positive diagnosis, and residing in Malaysia, Thailand or five African countries was negatively associated with a COVID-19 positive diagnosis. In addition, in the unadjusted analysis, older age was negatively, and female sex was positively associated with a COVID-19 positive diagnosis (see Table 2).

## Discussion

This study conducted among adults in nine LMIC from December 2020 to February 2021 showed that the COVID-19 testing uptake was 40.3% and that 7.3% tested SARS-CoV-2 positive, which is higher than in a large community survey in Canada (3.3% of those who had undergone testing were tested positive) [2] and in Germany (0.46 to 1.82% of the population-based cohort were tested positive) [5]. The high proportion of COVID-19 testing uptake and the higher proportion of positive SARS-CoV-2 tests may be partially attributed to the high participation rate of students or workers in the health care sector (34.4%) but also because in LMIC testing is more likely to be done among symptomatic individuals or persons in contact with positive cases.

Using the Andersen’s health care utilization model, we found that predisposing factors (residing in Brazil, postgraduate education), enabling/disabling factors (urban residence, higher perceived economic status, being a student or worker in the health care sector, and moderate or severe psychological distress), and need factors (having at least one chronic condition) increased the odds of COVID-19 testing. Previous studies also found that several underlying health conditions, higher household income, and occupation (health care worker) were associated with COVID-19 testing [2, 3]. Persons with underlying health conditions such as obesities, diabetes and cardiovascular diseases are at increased risk for developing severe diseases and therefore tested when they develop COVID-19 like symptoms [25]. People with higher education and higher economic status may have better access to health information, such as the importance of knowing one’s COVID-19 status. Unlike previous studies that found an association between older age, male sex, and uptake of COVID-19 testing [2, 3], we did not find age and sex differences in the uptake of COVID-19 testing. It is interesting to note the demographic differences in COVID-19 testing in LMICs, compared with previous studies conducted in Canada and the UK [2, 3]. Perhaps there was no concerted effort to test the elderly among the LMIC countries, unlike in Canada where long-term care facilities contributed significantly to COVID-19 mortality, necessitating the mass testing of elderly individuals [26]. Compared to Brazil, all other study countries had a lower uptake of COVID-19 testing. High COVID-19 testing was also observed among health care workers in Brazil (73.6%) [27]. This high uptake of testing in Brazil is explained by the high COVID-19 disease burden in Brazil and the widespread use of COVID-19 rapid antigen tests [28, 29].

Regarding the odds of COVID-19 positive diagnosis among all persons tested, residing in Bangladesh, and moderate to severe psychological distress were positively associated with COVID-19 positive diagnosis and residing in Malaysia and Thailand and higher education were negatively associated with a COVID-19 positive diagnosis. In the UK biobank data study, ethnicity (non-Whites) and lower education were associated with COVID-19 positive diagnosis [3]. However, both in this study and the UK biobank data study among tested persons, no significant association between comorbidities or health risk factors with a COVID-19 positive diagnosis was found [3].

The association between psychological distress and COVID-19 positive diagnosis has also been found among COVID-19 survivors in China [30] and may be attributed to stress reactions following the COVID-19 positive diagnosis potentially leading to long-term mental disorders [30]. In another study in China, depressive symptoms were more prevalent in COVID-19 patients compared to non-COVID-19 controls and were associated with an increase in inflammation markers [31]. Consequently, COVID-19 survivors should be screened for stress disorder, anxiety, and depression regularly to identify those with psychological distress for timely intervention [30]. The lower odds of COVID-19 positive diagnosis, among those with higher education can be explained by the significantly higher rate of COVID-19 testing among those with higher compared to those with lower education. Furthermore, it is possible that people with higher education and more access to quality information are more likely to take up vaccination, follow COVID-19 prevention measures and hence are less likely to become infected [32, 33]. The higher rate of COVID-19 positive diagnosis in Bangladesh may be explained by a higher prevalence of COVID-19 pandemic in Bangladesh (15.1%) [12], and the reluctance of people to get tested for COVID-19, when they have no or only moderate COVID-19 symptoms [34]. The lower rate of COVID-19 positive diagnosis in Malaysia may be attributed to a lower average test positive ratio and testing rate (4.3% and 0.8 tests per 1000 population) [11]. In Thailand the low rate of COVID-19 positive diagnosis may be attributed to a low prevalence of COVID-19 infection (7.5% among hospital patients) [13], and a low-test positive ratio (1.3, January–March 2021) [35], and a high compliance to COVID-19 preventive measures in Thailand [36].

The odds of a COVID-19 positive diagnosis among all individuals tested or not tested found increased with urban residence, higher perceived economic status, being a student or worker in the health sector, moderate or severe psychological symptoms, and not residing in Malaysia, Thailand or five African countries. We did not find a significant association between older age and having comorbidities and a COVID-19 positive diagnosis, as found in the Canadian study [2]. Other studies have likewise found that those who reside in urban areas are more likely to contract the COVID-19 virus compared to rural areas and small towns [37, 38]. This has been attributed to population density and the occurrence of large gatherings more common in urban areas, which accelerated the disease transmission through respiratory droplets in crowded conditions [39]. The higher odds of those who have higher perceived economic status being COVID-19 positive may be attributed to their higher likelihood of being tested, as was found in this study and in another study in New York City [40]. These results may point to the disparity in healthcare access, in which individuals from a higher socioeconomic status have better chances of being tested positive and therefore able to seek treatment. Finally, it is not surprising that workers and students in the healthcare sectors had higher odds of being tested COVID-19 positive due to their higher exposure to the COVID-19 virus.

Like the UK study [3], we found that having comorbidities increased the odds of COVID-19 testing but not with the risk of testing positive. This finding may suggest that having comorbidities may assist in predicting the risk of developing COVID-19 symptoms, and therefore the probability of getting a COVID-19 test [3]. In a study in Bangladesh, underlying health conditions/non-communicable diseases triggered factors for anxiety and depression symptoms. This worry/ fear might be responsible for a higher uptake in testing [41].

Higher education was found associated with COVID-testing uptake, but within the tested population lower education predicted a COVID-19 positive diagnosis. Individuals with lower education may be involved in manual or outdoor jobs which expose them to crowded working conditions (such as in factories), compared to those who have a higher education who may have the opportunity to work from their homes, and have more possibilities to adapt their working conditions to the pandemic situation [42, 43]. In addition, adherence to preventive measures has also been found to be lower among individuals with lower educational attainment [43]. Comparing COVID-19 positive diagnosis among those who tested and the whole sample, including those that had not tested, we found similarities on country, age, sex, living status, chronic conditions, not adhering to four COVID-19 preventive measures, correct COVID-19 knowledge, psychological distress and worry/fear about being (re)infected with COVID-19, but we also found differences in terms of higher education negatively associated with COVID-19 positive diagnosis within the tested population, and an association between urban residence, higher perceived economic status, being a student or worker in the health care sector and COVID-19 positive diagnosis in the whole study population. Health care workers have been identified as a high-risk group for contracting and spreading the COVID-19 virus, due to exposure to symptomatic and asymptomatic patients in healthcare settings, a lack of adherence to preventive measures, and the lack of personal protective equipment [4].

Although a high proportion (51.7%) of participants were very or extremely worried about being (re)infected with COVID-19, this translated only in the unadjusted analysis to higher odds of COVID-19 testing uptake, while higher psychological distress was associated with higher COVID-19 testing, and more severe psychological distress with a COVID-19 positive diagnosis. Individuals who have contracted the virus were found to exhibit more depression and anxiety symptoms in other studies. In a study in Italy among adults surviving COVID-19 infection, 31% presented depression symptoms and 42% anxiety symptoms [44]. It is possible that the psychological distress increased after COVID-19 positive diagnosis, but since survey was only a cross-sectional study, we are not able to determine the direction of the relationship between psychological distress, testing and testing positive. However, within the tested population, psychological distress was inversely associated with a COVID-19 negative diagnosis (analysis not shown).

### Study limitations

Our study respondents cannot be considered representative of the general population in the study countries since respondents needed to have had access to the internet to participate in the online survey. We acknowledged this limitation of this study and do believe that studies like this one help in health surveillance actions, which directly impact the evolution of the pandemic in a country and the selection of more assertive preventive measures. Moreover, self-reports, including the outcome variable COVID-19 testing, may be influenced by recall bias and social desirability. Another potential limitation was the poor quality of certain data, e.g., the informational quality of information provided by health care personnel and other information sources. In addition, cultural differences between countries and between regions of the same country can be a limitation in relation to the quality of the information. We also have no information on the type of test used for COVID-19 testing, limiting the accuracy and authenticity of the test results. Some variables, such as COVID-19 symptoms, previous use of health care services, obesity, and smoking, that have been found affecting COVID-19 testing uptake [2, 3] were not assessed in this survey and should be included in future studies.

## Conclusions

This study among adults across nine LMICs reported a high prevalence of COVID-19 testing. Factors associated with COVID-19 testing included predisposing factors (residing in Brazil, postgraduate education), enabling/disabling factors (urban residence, higher perceived economic status, being a student or worker in the health care sector, and moderate or severe psychological distress), and need factors (having at least one chronic condition). Identified predisposing and enabling or disabling factors can be used to design programmes to improve COVID-19 testing uptake. Access to testing needs to be increased for persons living in Africa and similar resource poor settings. In addition, COVID-19 testing programs need to target persons of lower economic status and of lower education level who currently less tested but most at risk for COVID-19 infection.

## Data Availability

Data are available upon reasonable request. Data are available on the International Consortium (International Citizen Project COVID-19 (ICPcovid): http://www.icpcovid. com) website and could be used by other investigators on request. De-identified participant data are available from Joseph Nelson Siewe Fodjo at Email: josephnelson.siewefodjo@uantwerpen.be

## Abbreviations

LMCI: Low- and middle-income countries
PHQ: Patient Health Questionnaire
PM: particulate matter
